# Petrophilic, Fe(III) Reducing Exoelectrogen *Citrobacter* sp. KVM11, Isolated From Hydrocarbon Fed Microbial Electrochemical Remediation Systems

**DOI:** 10.3389/fmicb.2018.00349

**Published:** 2018-03-12

**Authors:** Krishnaveni Venkidusamy, Ananda Rao Hari, Mallavarapu Megharaj

**Affiliations:** ^1^Centre for Environmental Risk Assessment and Remediation (CERAR), University of South Australia, Mawson Lakes, SA, Australia; ^2^CRC for Contamination Assessment and Remediation of the Environment (CRCCARE), Mawson Lakes, SA, Australia; ^3^Division of Sustainable Development, Hamad Bin Khalifa University, Education City, Doha, Qatar; ^4^Global Centre for Environmental Remediation (GCER), Faculty of Science, The University of Newcastle, Callaghan, NSW, Australia

**Keywords:** petrophilic, electroactive biofilms, *Citrobacter* sp. KVM11, iron reducing, extracellular electron flow, microbial electrochemical remediation systems, hydrocarbonoclastic potential

## Abstract

Exoelectrogenic biofilms capable of extracellular electron transfer are important in advanced technologies such as those used in microbial electrochemical remediation systems (MERS) Few bacterial strains have been, nevertheless, obtained from MERS exoelectrogenic biofilms and characterized for bioremediation potential. Here we report the identification of one such bacterial strain, *Citrobacter* sp. KVM11, a petrophilic, iron reducing bacterial strain isolated from hydrocarbon fed MERS, producing anodic currents in microbial electrochemical systems. Fe(III) reduction of 90.01 ± 0.43% was observed during 5 weeks of incubation with Fe(III) supplemented liquid cultures. Biodegradation screening assays showed that the hydrocarbon degradation had been carried out by metabolically active cells accompanied by growth. The characteristic feature of diazo dye decolorization was used as a simple criterion for evaluating the electrochemical activity in the candidate microbe. The electrochemical activities of the strain KVM11 were characterized in a single chamber fuel cell and three electrode electrochemical cells. The inoculation of strain KVM11 amended with acetate and citrate as the sole carbon and energy sources has resulted in an increase in anodic currents (maximum current density) of 212 ± 3 and 359 ± mA/m^2^ with respective coulombic efficiencies of 19.5 and 34.9% in a single chamber fuel cells. Cyclic voltammetry studies showed that anaerobically grown cells of strain KVM11 are electrochemically active whereas aerobically grown cells lacked the electrochemical activity. Electrobioremediation potential of the strain KVM11 was investigated in hydrocarbonoclastic and dye detoxification conditions using MERS. About 89.60% of 400 mg l^-1^ azo dye was removed during the first 24 h of operation and it reached below detection limits by the end of the batch operation (60 h). Current generation and biodegradation capabilities of strain KVM11 were examined using an initial concentration of 800 mg l^-1^ of diesel range hydrocarbons (C9-C36) in MERS (maximum currentdensity 50.64 ± 7 mA/m^2^; power density 4.08 ± 2 mW/m^2^, 1000 ω, hydrocarbon removal 60.14 ± 0.7%). Such observations reveal the potential of electroactive biofilms in the simultaneous remediation of hydrocarbon contaminated environments with generation of energy.

## Introduction

Electrochemical oxidation by electroactive biofilms is vital to the performance of microbial electrochemical remediation systems (MERS) and enhanced removal of contaminants. Such remediation systems transform the chemical energy available in organic pollutants into electrical energy by capitalizing on the biocatalytic potential of electroactive communities (Morris and Jin, 2012; Venkidusamy et al., 2016). These systems offer a unique platform to study the electro-microbial process involved in bioremediation of oil pollutants (Venkidusamy et al., 2016) and heavy metals (Qiu et al., 2017; Wang et al., 2017), etc., The electroactive biofilms are those that have the capabilities of extracellular electron flow (EET) to degrade substrates that range from easily degradable natural organic compounds to xenobiotic compounds such as petroleum hydrocarbon (PH) contaminants (Venkidusamy et al., 2016; Zhou et al., 2016). Such biofilms can be formed by a single bacterial species (pure strain) (Venkidusamy and Megharaj, 2016a,b) or by multiple bacterial species (mixed culture) (Morris et al., 2009). The dominant view, until recently is that multiple bacterial species are better suited for its commercial applications (Chae et al., 2009), while the single bacterial species are selected to study their physiology and electrochemical performance (Xing et al., 2008; Zhi et al., 2014; Venkidusamy and Megharaj, 2016a,b; Qiu et al., 2017).

Petrochemical products are widespread contaminants that have long been of serious concern for environmental public health. Of these, diesel range hydrocarbons (DRH) became the most encountered environmental pollutants due to its increasing anthropogenic activities. Microbial removal of these DRH compounds is claimed to be an efficient, economical and versatile alternative to the established physicochemical treatments that are prone to cause recontamination by secondary contaminants (Hong et al., 2005; Megharaj et al., 2011). The biodegradation of these compounds at the soil surface has been well documented for a century (Atlas, 1991; Chaillan et al., 2004) whereas sub-surface biodegradation awaits further research on deeper insights into the metabolic activities involved and the extent and rate of hydrocarbon degradation (Röling et al., 2003). Such anaerobic, hydrocarbon contaminated reservoirs are dominated by obligate and facultative “petrophilic” (microorganisms capable of degrading hydrocarbons (Mandri and Lin, 2007) microbial communities (Singh et al., 2014). These microbial communities can adjust their metabolism based on the availability of terminal electron acceptors and can have more complex enzymatic systems involved in the degradation of contaminants. However, the rate of microbial utilization of these PH compounds is very slow especially under anaerobic environments where the availability of relevant electron acceptors is limited (Widdel et al., 2006; Foght, 2008; Morris et al., 2009). Emerging technologies on the removal of such recalcitrant contaminants using electrodes and biofilms are gaining new interest in their applications due to its enhanced remediation (Venkidusamy et al., 2016) and continuous sink for electron acceptors such as electrodes in an economical way (Wang and Ren, 2013; Li et al., 2014).

To date, however, the mechanisms of EET are well characterized in iron reducing microbial strains from a couple of dominant model taxa such as *Geobacter* (Bond and Lovley, 2003; Reguera et al., 2005) and *Shewanella* (Kim et al., 2002; El-Naggar et al., 2010), the delta-gamma subgroups of Proteobacteria. Beyond these model taxa, however, electrochemical enrichments and 16S rRNA gene sequencing-based studies from diverse environments have shown the presence of physiologically and phylogenetically diverse, electroactive microbial communities on fuel cell electrodes. These microbial communities include the members of Alphaproteobacteria (Zuo et al., 2008), Betaproteobacteria (Chaudhuri and Lovley, 2003), Gammaproteobacteria (Kim et al., 2002), Deltaproteobacteria (Holmes et al., 2006), and Firmicutes (Wrighton et al., 2008). Of these, *Gammaproteobacteria* was the dominant class, and several bacterial strains from this class have been isolated either from electrochemical systems fed with wastewater or defined carbon sources and their physiological roles have been studied (Choo et al., 2006; Logan and Regan, 2006). Many of these exoelectrogens are dissimilatory Fe(III) reducers that possess the ability to reduce the insoluble Fe(III) in different environments such as sediments and groundwater aquifers (Caccavo et al., 1994; Coates et al., 1999; Kunapuli et al., 2010; Venkidusamy et al., 2015). For instance, *Geobacter sulfurreducens*, a dissimilatory Fe(III) reducer isolated from PH contaminated aquifers showed maximum current density of 65 mA/m^2^ using acetate as a carbon source (Bond and Lovley, 2003). Recent studies have shown the diversity of different genetic groups of Fe(III) reducers such as, *Thermoanaerobacter pseudoethanolicus* (Lusk et al., 2015), *Thermincola ferriacetica* (Parameswaran et al., 2013), *Geoalkalibacter* sp. (Badalamenti et al., 2013) *Clostridium butyricum* (Park et al., 2001) etc., which can transfer electrons to solid phase electron acceptors with co-degradation of recalcitrant contaminants (Kunapuli et al., 2010). For instance, *Rhodopseudomonas palustris* strain RP2, a dissimilatory Fe(III) reducer isolated from PH fed MERS has been shown to produce a maximum current density of 21 ± 3 mA/m^2^; with simultaneous removal of 47 ± 2.7% in MERS within 30 days (Venkidusamy and Megharaj, 2016b). It is important to note that the microbial community composition is divergent in MERS (Morris et al., 2009; Venkidusamy et al., 2016) fed with contaminants such as petrochemicals and the physiology of such microbial populations remains to be explored. Recent research on removal of such recalcitrant contaminants using MERS is gaining interest in its practical applications by employing selected bacterial species for sub-surface PH bioremediation (Morris et al., 2009; Venkidusamy et al., 2016). This makes the identification of such bacterial population with functions of electrode respiration and PH degradation, fundamental to investigating the contaminant removal processes in MERS systems.

Our study was motivated by both apparent nature of Fe(III) reducing electroactive biofilms and contaminant degradation that represents the possibilities of microbe-electrode-contaminant interactions in MERS systems. In our laboratory, hydrocarbon fed MERS have been successfully demonstrated for the enhanced removal of PH contaminants (Venkidusamy et al., 2016). The subsequent isolation and characterization of single bacterial species from the exoelectrogenic biofilms of PH fed MERS suggests that isolated bacterial strains gained an advantage of extracellular electrode respiration (Venkidusamy et al., 2015; Venkidusamy and Megharaj, 2016a) and Fe(III) reduction (Venkidusamy and Megharaj, 2016b) as reported earlier. In this study, we report one such Fe(III) reducing bacterial strain phylogenetically related to *Citrobacter* genus and designated as *Citrobacter* sp. KVM11. The strain was found to be a facultative anaerobe. The electrochemical activity was determined by using fuel cell experiments (in different conditions) and voltammetry studies. Here, we show the existence of current generation and biodegradation capabilities by the strain KVM11 in PH fed, and azo dye fed MERS for the first time. Our findings contribute to the emerging view that MERS has great potential to offer a new route to the sustainable bioremedial process of contamination with simultaneous energy recovery by its electroactive biofilms.

## Materials and Methods

### Bacterial Strain

The bacterial strain used in the study was isolated from the electrode attached biofilm of a hydrocarbon-fed electrochemical reactors through serial dilution techniques. The initial source of inoculum for the PH fed MERS was a mix of PH contaminated groundwater and activated sludge. These MERS were operated in a fed-batch mode (30 days) over a period of 12 months with a PH concentration of 800 mg l^-1^ as described earlier (Venkidusamy et al., 2016). Bacterial cells from the electrode biofilm were extracted into a sterile phosphate buffer and shaken vigorously to separate the cells from the electrode. The extracted cell suspensions were serially diluted and plated onto modified Hungate’s mineral medium (Hungate, 1950) containing acetate (20 mM) as an electron donor and ferric(III) citrate as the electron acceptor (10 mM) and incubated anaerobically in a glove box (Don Whitley Scientific, MG500, Australia) for a period of 3 weeks. Single colonies were selected and transferred to Luria-Bertani (LB) agar plates. Media used throughout the study were Luria-Bertani medium (Sambrook et al., 1989) and Bushnell Hass medium (Hanson et al., 1993). A chemically defined medium supplemented with Wolfe’s trace elements and vitamins was used in the microbial electrochemical studies as previously described (Oh et al., 2004). One liter of growth medium contains (g l^-1^) KCl 0.13, Na_2_HPO_4_ 4.09, NaH_2_PO_4_ 2.544, NH_4_Cl 0.31. The pH of the medium was adjusted to 7.0 ± 0.2 and further fortified with Wolfe’s trace elements and vitamins. The purified strain was stored in glycerol: Bushnell Hass broth and glycerol: Luria-Bertani broth (1:20) at -80 °C. Biolog-GN2 (Biolog Inc., United States) plates were used to determine the utilization of various carbon sources under anaerobic conditions according to the manufacturer’s instructions.

### Iron (III) Reduction Experiments

Fe(III) citrate (10 mM) served as the terminal electron acceptor in anaerobic iron reduction experiments. The cells were grown in Wolfe’s medium using acetate (20 mM) supplemented with trace elements and vitamins (Lovley and Phillips, 1988a). All procedures for Fe(III) reduciton experiments, from medium preparation to manipulating the strain were performed using standard anaerobic conditions. All solution transfers and samplings of the cell cultures were trasnferd under anaerobic (10% hydrogen, 10% carbon dioxide, and 80% nitrogen) (Don Whitley Scientific, MG500, Australia) conditions using syringes and needles that had been sterlized. Fe(III) reduction was determined using the ferrozine assay (Lovley and Phillips, 1988b). The bacterial suspension was added to a pre-weighed vial containing 0.5 M HCl. HCl extracted samples were added to 5 ml of ferrozine (1 g l^-1^) in 50 mM HEPES buffer. The filtered samples were then analyzed in a UV-Vis spectrophotometer (maxima@λ562 nm) to quantify the Fe(II) formation as previously described (Lovley and Phillips, 1988b).

### Microscopy

Bacterial samples for transmission electron microscopy were fixed in an electron microscopy fixative (4% paraformaldehyde/1.25% glutaraldehyde in PBS, + 4% sucrose, pH-7.2) and washed with buffer. Samples were postfixed in 2% aqueous osmium tetroxide. They were dehydrated in a graded series of ethanol and then infiltrated with Procure/Araldite epoxy resin. Blocks were polymerized overnight at 70°C. Sections were cut on a Leica UC6 Ultramicrotome using a diamond knife, stained with uranyl acetate and lead citrate and examined in an FEI Tecnai G2 Spirit Transmission Electron Microscope. The samples were also prepared using a heavy metal negative staining method involving phosphotungstic acid. The electrode samples were also fixed and prepared as described earlier (Venkidusamy et al., 2016). The dried brush samples were examined with a scanning electron microscope (Quanta FEG 450, FEI) at an accelerating voltage of 20 kV.

### Phylogenetic Analysis

The genomic DNA of the bacterial strain was extracted using the UltraClean microbial DNA isolation kit (MO BIO, CA) following the manufacturer’s instructions. The universal primers E8F (5′-AGAGTTTGATCCTGGCTCAG3′) and 1541R (5′AAGGAGGTGATCCANCCRCA 3′) were used to amplify 16S rRNA gene according to the procedure by Weisburg et al. (1991). The polymerase chain reaction (PCR) mix of 50 μl contained the following: 10 μl of Gotaq 5X buffer, 2.0 μl of MgCl_2_ (25 mM), 1 μl of dNTP mix (1 mM), 2 μl of each primer (100 mM), 10–15 ng of purified DNA, and 2.5 U Taq DNA polymerase (Promega, Australia). PCR amplification was performed with an initial denaturation for 5 min, followed by 35 cycles of the 60 s at 94°C, 30 s of annealing at 40–60°C, 60 s of extension at 72°C, and a final extension at 72°C for 10 min, using a Bio-Rad thermal cycler. The PCR products were purified via the UltraClean PCR clean-up kit (Mo Bio, CA) following the manufacturer’s instructions, and sequenced by the Southern Pathology Sequencing Facility at Flinders Medical Centre (Adelaide, South Australia). *In silico* analysis of 16S rRNA gene sequences was done by using the blast programs to search the GenBank and NCBI databases^1^. The highest hit for the isolate KVM11 was used for ClustalW alignment and phylogenetic relationship generation. The neighbor-joining tree was constructed using the molecular evolutionary genetic analysis package version 5.0 (MEGA 5.0) based on 1000 bootstrap values (Tamura et al., 2011).

### Assessment of Electrochemical Activity and Biodegradation Potential

Experiments were also performed to evaluate the possible candidate electroactive bacterial strain by *in vivo* decolourization assay using diazo dyes as described earlier (Hou et al., 2009). Experiments were carried out both aerobically and anaerobically using 20 ml of nutrient broth (Peptone-15g; D(+)glucose-1g; Yeast extract-3g; NaCl-6g) with a concentration of 400 mg l^-1^ of an azo dye, Reactive Black5 (RB5). The dye degradation was monitored by observing the decrease in absorbance of suspension at 595 nm under a UV-visible spectroscopy system (Agilent model 8458) and visible color change. All decolorization studies were maintained in triplicate for each experiment, and the activity was expressed as percentage degradation. The hydrocarbon degradation potential of strain KVM11was evaluated by measuring the reduction of metabolic indicators such as dichlorophenol indophenol (DCPIP) and tetrazolium salts (Pirôllo et al., 2008).

### Fuel Cell Experiments

#### MFC Construction and Operation

Single chamber bottle MFCs were made from laboratory bottles with a capacity of 320 ml as previously described by Logan (2008) (Supplementary Figure S1). The liquid volume of the chamber was 280 ml. Anodes were carbon paper or graphite fiber brushes of 5 cm in diameter and 7 cm in length. The graphite brushes were treated as previously described (Feng et al., 2010). The cathode was made using flexible carbon cloth coated with a hydrophobic PTFE layer with added diffusional layers on the air breathing side whereas the hydrophilic side was coated using a mixture of Nafion perfluorinated ion exchange ionomer binder solution, carbon and platinum catalyst (0.5 g of 10% loading (Cheng et al., 2006). The surface area of the anodic electrode was calculated using a porous analyser, and the cathode’s total projected area was 15.6 cm^2^. All the electrodes were thoroughly rinsed in deionized water and stored in distilled water prior to use. The electrodes were attached using copper wire, and all exposed surface areas were covered by non-conductive epoxy resin (Jay Car, Australia). All the reactors were stream sterilized in an autoclave before use. The bacterial cell suspension was prepared by pipetting bacterial cells (cell density, 1% 1OD culture) into a sterile centrifuge tube by centrifugation at 4500 rpm for 20 min. The supernatant was decanted, and the pellet containing cells were washed and resuspended in PBS before inoculation into MERS. The anode compartment was fed with 50 mM PBS (neutral pH) and salts as stated earlier (Oh et al., 2004). Acetate and citrate were used as carbon sources (1 g/L) in fuel cell experiments. The anode chamber was purged with nitrogen gas to maintain anaerobic conditions. The anolyte was agitated using a magnetic stirrer operating at 100 rpm. Open circuit (OC) MFC studies were also carried out and then switched to the closed circuit with a selected external load (R-1000 ω unless stated otherwise). Solutions were replaced under anaerobic chamber when the voltage dropped to a low level (≤10 mV). All the reactors were maintained at room temperature in triplicates.

#### Electrochemical Analysis

Bacterial cells grown in Fe(III) citrate liquid cultures were harvested and used for electrochemical studies. The direct electrode reaction of the cells was examined using cyclic voltammetry (CV) using a conventional three electrode electrochemical cell with a 25 ml capacity. Cyclic voltammograms of the bacterial suspension were obtained using a potentiostat (Electrochemical analyser, BAS 100B, United States) connected to a personal computer. Cells were examined under nitrogen atmosphere at 25°C. A glassy carbon working electrode (3 mm, diameter, MF-2012, BAS) and silver/silver chloride reference electrode (MW-4130, BAS) and platinum counter electrode (MW-4130, BAS) were used in a conventional three-electrode system. The working electrodes were polished with alumina slurry on cotton wool followed by ultra-sonic treatments for about 10 min. The electrochemical cells were purged with nitrogen gas for 15 min before each measurement. The scan rate was 5 mV s^-1^ with a potential range from -800 to 800 mV.

#### Electrobioremeditaion Experiments

Hydrocarbon biodegradation potential was monitored under MERS conditions using 1% (1 OD) inoculum and 800 mg l^-1^ of DRH as a sole source of carbon. All cell cultures were maintained in triplicate for each experiment. Reactive Black 5 was used as sole source of energy in dye degradation experiments using the strain at a concentration of 50 mg l^-1^ in MFC studies. LB medium was used in decolorization studies with an external load of 1000 ω. MFCs were operated in a fed-batch mode until the voltage fell to a low level (≤10 mV) and then the anolyte solution was replaced under anaerobic (10% hydrogen, 10% carbon dioxide, and 80% nitrogen) (Don Whitley Scientific, MG500, Australia) conditions. All procedures for degradation experiments, from medium preparation to manipulating the strain were performed using standard anaerobic conditions. OC and abiotic controls (AC) were prepared for each set of biodegradation experiments. All the reactors were maintained at room temperature in triplicates.

### Analytical Methods and Calculations

Fe(III) reduction was monitored by measuring Fe(II) production using the ferrozine method (Lovley and Phillips, 1988b). The fuel cells were continuously monitored for voltage generation across the resistor using a digital multimeter (Keithley Instruments, Inc., Cleveland, OH, United States) linked to a multi-channel scanner (Module 7700, Keithly Instruments, United States). Unless otherwise stated, all the MFC cycles were loaded with an external resistance of 1000 ω. Current (I) and power (P) were calculated as previously described (Logan, 2008) and normalized to the cathode surface area (mW/m^2^). Graphite fiber surface area was also measured using a Brunauer-Emmett-Teller (BET) isotherm (Mi micrometrics, Gemini V, Particle and Surface Science Pty Ltd). DRH concentrations were measured by GC-FID using a HP-5 capillary column (15 m length, 0.32 mm thickness, 0.1 mm internal diameter) following the USEPA protocol (USEPA, 1996). The resulting chromatograms were analyzed using Agilent software (GC-FID Agilent model 6890) to identify the hydrocarbon degradation products. Chemical oxygen demand was measured by COD analyzer using effluent samples from the reactors reactors fed with acetate and citrate (Chemetrics, K-7365). Polarization curves were plotted by using various external loads with a range of 10 ω to open circuit. Coulombic efficiency (CE) was calculated at the end of the cycle from COD removal as previously described by Logan (2008).

### Nucleotides Accession Number

The 16S rRNA gene sequence obtained from this study has been deposited in the European nucleotide achieve database collections under the accession number of KY693675.

## Results and Discussion

### Strain Isolation, Phenotype, Phylogenetic Analysis and Taxonomy

**FIGURE 1:**
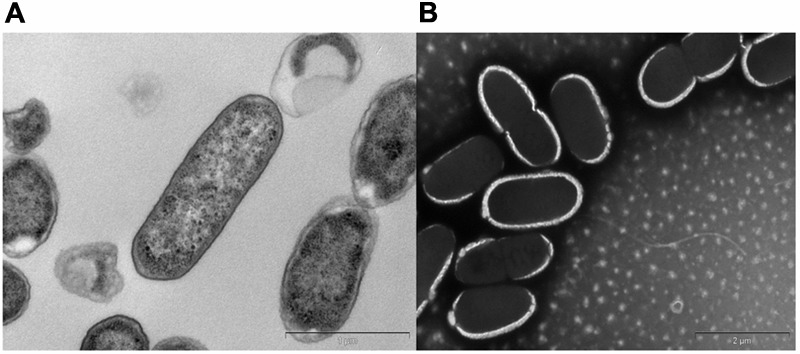
Transmission electron micrographs of *Citrobacter* sp. KVM11. Bacterial cells were fixed in electron microscopy fixative (4% paraformaldehyde/1.25% glutaraldehyde in PBS, + 4% sucrose, pH-7.2) and washed with buffer. **(A)** Transverse section of the polymerized cells of KVM11, bar scale 100 nm **(B)** Negatively stained cells of KVM11 with filaments, bar scale 200 nm. The samples were prepared using a heavy metal staining method involving phosphotungstic acid. Samples were examined in an FEI Tecnai G2 Spirit Transmission Electron Microscope.

**FIGURE 2:**
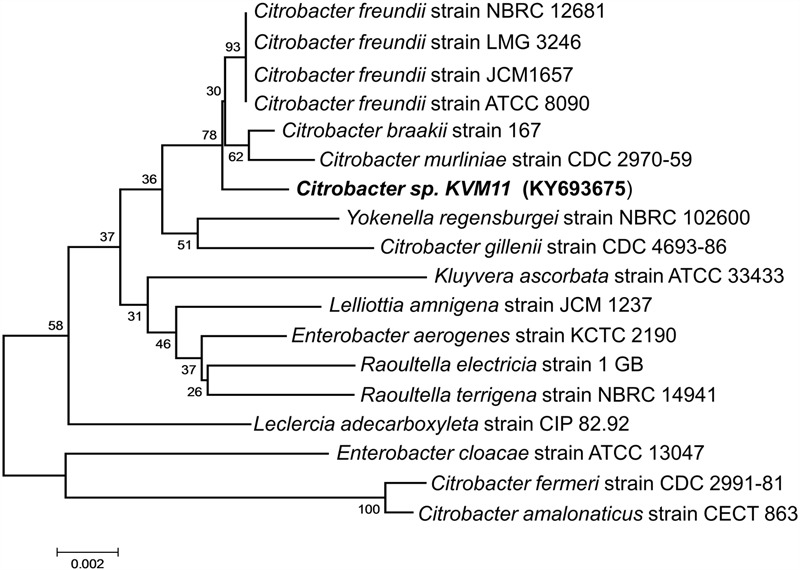
Phylogenetic tree based on 16S rRNA gene sequences showing the positions of the isolated *Citrobacter* sp. KVM11 and closest representatives of other *Citrobacter* sp. The sequences of *Citrobacter farmeri* and *C. amalonaticus* formed an outgroup sequence. The tree was constructed from 1,418 aligned bases using the neighbor-joining method. The number at nodes show the percentages of occurrence of the branching order in thousand bootstrapped trees. Scale bar represents 0.005 substitution per nucleotide position.

A bacterial strain designated KVM11 was isolated from PH fed MERS operated free of external mediators by serial dilution and plating techniques. Cultures with a single morphotype were obtained and found to be composed of double membrane bilayers (Gram-negative), short bacilli shaped (2–4 μM in length), facultatively anaerobic, motile using flagella in tufts or individual for its locomotion (**Figure 1**). Cell growth on LB medium produces creamy, translucent colonies with a shiny surface. Cell reproduction occurred via binary fission with two identical daughter cells. A 1418 bp (almost entire length) target fragment of 16S rRNA was amplified by PCR using a genomic DNA of strain KVM11 and 16S rRNA primers. Using this multiple alignment, the neighborhood phylogenetic tree was constructed as shown in **Figure 2**. The taxonomic position of strain KVM11 showed a close affiliation with the genus *Citrobacter* in the class of Gammaproteobacteria. The closest recognized relatives of this strain were *Citrobacter freundii* ATCC 8090, *C. freundii* strain NBRC 12681, *C. freundii* strain LMG 3246, *C. braakii* strain 167, *C. murliniae* strain CDC 2970-59 which shared 99% similarity in their 16S rRNA gene sequence. These *Citrobacter* sp. constitute one of the most diverse, known commensal inhabitants that colonizes a variety of aquatic environments, soil, sewage sludges and gastrointestinal tracts of both humans and animals (Wang et al., 2000; Narde et al., 2004).

### Physiological and Metabolic Properties

**FIGURE 3:**
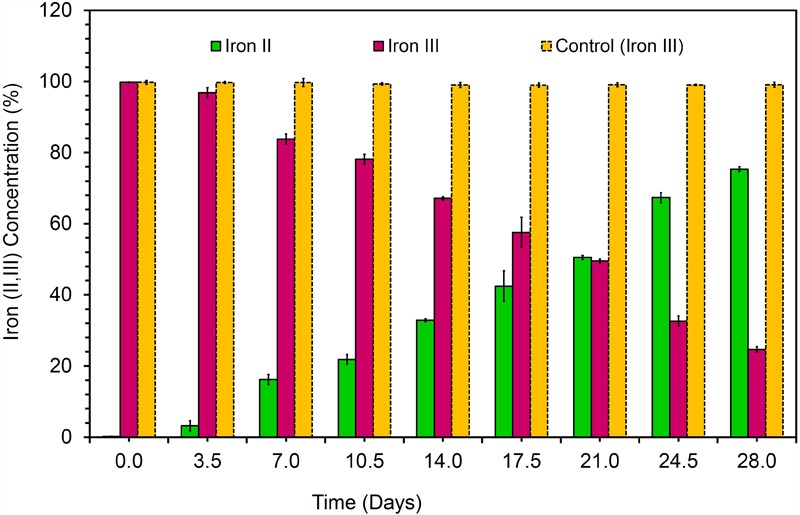
Dissimilatory Fe(III) oxide reduction in anaerobically incubated cells of strain KVM11 at designated intervals (Ferrozine assay, Yellow bar represents the percent of Fe(III) reduction in chemotropically grown control cells; Pink bar represents Fe(III) reduction in anaerobically incubated samples of KVM11; Green bar shows Fe(II) formation in incubated samples of KVM11. Cells were inoculated into an anaerobic vials containing growth medium, electron donor: 20 mM acetate and electron acceptor: 10 mM Fe(III).

The bacterial strain is a mesophile that typically grows at temperatures ranging from 25 to 37°C. The strain was negative for oxidase and positive for catalase. The bacterial strain KVM11 can grow based on environmental signals of aerobic and anaerobic heterotrophic mechanisms as reported earlier in other strains of this genus (Oh et al., 2003). The strain was shown to be capable of dissimilatory nitrate reduction through biochemical analysis as seen in a number of exoelectrogenic bacterial strains (Xing et al., 2010; Venkidusamy and Megharaj, 2016a). The cells were grown under anoxic, chemoheterotrophic conditions with Fe(III) citrate as a terminal electron acceptor to investigate the dissimilatory Fe(III) reduction trait. Fe(III) reduction was monitored by color change and hydroxylamine Fe(II) extraction assay. The color change of medium from pale yellow to dark greenish precipitate was observed in inoculated liquid cultures under anaerobic conditions. Their colonies were coated with Fe(II) precipitate as reported for other groups of exoelectrogens such as *Geobactor, Aeromonas* sp. and Fe(III) enriched samples (Pham et al., 2003; Chung and Okabe, 2009; Liu et al., 2016). Fe(III) reduction of 75.33 ± 0.70% was observed during 4 weeks of incubation with Fe(III) supplemented liquid cultures (**Figure 3**) whereas heat killed controls showed no reduction. Moreover, by the end of 36-day incubation there was a 90.01 ± 0.43% reduction of Fe(III). Abiotic loss of Fe(III) measured under each stage was less than 2%. Recent investigations have revealed the potential of using Fe(III) reducers in microbial electrochemical systems which include *Thermoanaerobacter pseudoethanolicus (*Lusk et al., 2015), *Thermincola ferriacetica* (Parameswaran et al., 2013), *Geoalkalibacter* sp., (Badalamenti et al., 2013) and *Clostridium butyricum* (Park et al., 2001). With regards to the *Citrobacter* strains, for example, *Citrobacter* sp. LAR-1 (Liu et al., 2016) and *C. freundii* Z7 (Huang et al., 2015) have also shown to be Fe(III) reducing exoelectrogens, although the rate of Fe(III) reduction is unknown. The strain KVM11 displayed a wide nutritional spectrum as highlighted by its utilization of various carbon sources under anaerobic conditions from its counterparts, *Citrobacter* sp. LAR-1 (Liu et al., 2016) *C. freundii* Z7 (Huang et al., 2015) (**Table 1**). However, the strain showed a different carbon source profile than the previously reported strains of *Citrobacter* with regards to its ability to assimilate a range of substrates including alanine, phenylalanine, adonitol, aminobutyric acid, lactose, etc., (Brenner et al., 1993, 1999). The increased cell content seen in cell cultures supplemented with glucose and pyruvate under anoxic conditions in the absence of electron acceptors depicts that the strain is also capable of fermentation as previously reported (Xu and Liu, 2011). Thus, this strain shares general characteristics with the *Citrobacter*, a genus of Enterobactereriaceae (Garrity et al., 2005).

**Table 1:** Phenotype and metabolic properties of *Citrobacter* sp. KVM11.

Particulars	*Citrobacter* sp. KVM11	*Citrobacter* sp. LAR-1 (Liu et al., 2016)	*Citrobacter freundii* Z7 (Huang et al., 2015)
Cell length (μm)	2–4	2–4	1–5
Cell shape	short bacilli	short bacilli	short bacilli
Motility	+	+	+
Optimum pH	6.5–7.2	6.5–7.0	6.5–7.0
Exoelectrogenic behavior	+	+	+
Nitrate reduction	+	NA	NA
Denitrification	+	NA	NA
Petroleum hydrocarbons	+	NA	NA
Catalase	+	NA	NA
Indophenol oxidase	-	NA	NA
Glucose fermentation	+	+	+
Lactose fermentation	+	+	+
Tween hydrolysis	-	NA	NA
Urea hydrolysis	+	+	NA
Acetate	+	+	+
Arabinose	+	NA	NA
Adonitol	+	NA	NA
Cellobiose	+	+	NA
Fructose	+	NA	NA
Maltose	+	NA	NA
Mannitol	-	+	NA
Xylose	-	+	NA
Citrate	+	+	+
Rhamnose	+	+	+
Gluconate	+	+	NA
N-Acetylglucosamine	-	+	NA
Sulfide production	+	NA	NA

*(+): Positive reaction; (-): Negative reaction; Not available: (NA).*

### Hydrocarbon Degradation Assays

The petrochemical degradation potential of strain KVM11 was assessed through a preliminary investigation of hydrocarbon consumption, a concomitant increase in biomass and reduction of redox electron acceptors such as DCPIP and tetrazolium indicators. The strain KVM11 discolored the redox indicator from the blue to violet during the first 24 h and complete discoloration was observed by the end of 120 h when DRH was the sole carbon and energy source. The respiratory reduction of tetrazolium salts is another criterion employed by many researchers (Olga et al., 2008; Pirôllo et al., 2008) to determine the dehydrogenase activity of hydrocarbon degrading bacterial strains. The formation of a red precipitate from the tetrazolium was observed while the AC remained unchanged. Upon reduction of this salt, the color changed to red due to the formation of insoluble formazan by the production of superoxide radicals and electron transport in the bacterial respiratory chain (Haines et al., 1996). It is evident from the screening assays that the strain KVM11 possess the hydrocarbon biodegradation potential by involving redox reactions in which electrons are donated to terminal electron acceptors during the cell respiration as previously described in other hydrocarbonoclastic strains (Olga et al., 2008; Venkidusamy and Megharaj, 2016a,b). The reduction of a lipophilic mediator such as DCPIP (blue to colorless) coupled with the formation of oxidized products showed that the biodegradation had been carried out by metabolically active cells involving growth, and not by adsorption to cells associated with the water-carbon interface (Kubota et al., 2008). It is of interest that, the aerobic mineralization of PH as its sole carbon source highlights the cosmopolitan presence of *Citrobacter* sp. in petrochemical contaminated sites (Singh and Lin, 2008; Morris et al., 2009).

### Assessment of Electrochemical Activity

**FIGURE 4:**
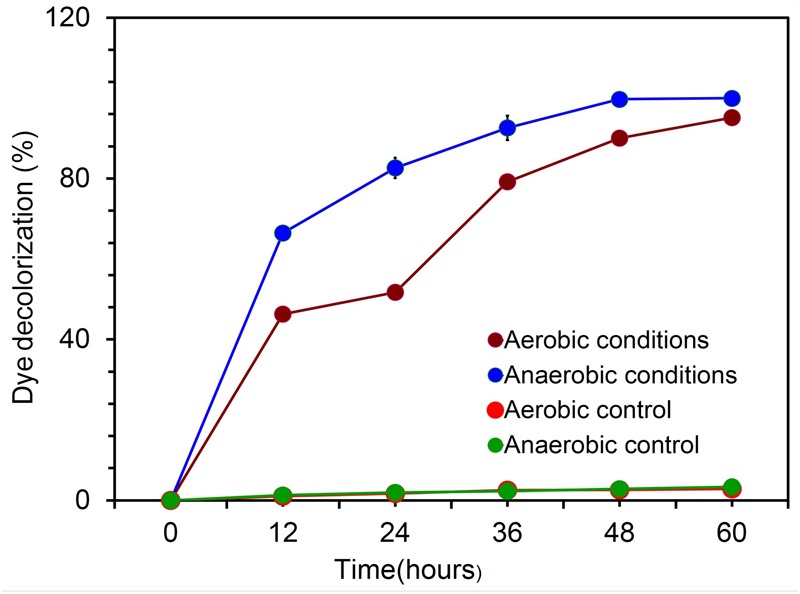
Biodecolourization of diazoic dye RB5 by the strain *Citrobacter* sp. KVM11 under aerobic and anaerobic environments. Experiments were carried out both aerobically and anaerobically using 20 ml of nutrient broth (Peptone-15g; D(+)glucose-1g;Yeast extract-3g;NaCl-6g) with a concentration of 400 mg l^-1^ of an azo dye, Reactive Black5 (RB5). Blue line represents anaerobically grown strain KVM11 cells; Green line shows control samples kept under anaerobic conditions; Maroon line represents aerobically grown strain KVM11 cells; Red line shows control samples kept under aerobic conditions.

The characteristic feature of diazo dye decolorization was used as a simple criterion for evaluating the possible electrochemical activity in microbial candidate in the present study as stated earlier (Hou et al., 2009). To assess the electrochemical activity of the isolate, aerobic and anaerobic cultures were grown in nutrient broth supplemented with 400 mg l^-1^ of Reactive Black5. The complete disappearance of the characteristic absorption peak at the region of bbbmax (597) and simultaneous decolorization of dye were observed after 48 h in aerobically grown samples. The highest rate of decolorization of azoic dye was observed at the end of 60 h under aerobic incubations (95.03%, **Figure 4**), whereas this tended to be faster under anaerobic conditions. RB5 azoic dye was almost completely decolorized (99.73%) in 48 h by *Citrobacter* sp. strain KVM11 under anaerobic conditions (**Figure 4**) as reported in another exoelectrogenic strain of *Shewanella* sp. (Pearce et al., 2006). This is in agreement with the previous studies on the assessment of electrochemically active microbial strains using azo dye fed MFC arrays (Hou et al., 2009). The blue pigmented dead cell pellet from the heat-killed cells in control showed a passive adsorption of dye, whereas colorless cell pellets obtained from the living cultures demonstrated the reduction of the RB5 indicator. Dye decolorization occurs because of a reductive electrophilic cleavage of the chromophore, a functional group of azo linkage, by biocatalysts as reported earlier (Sun et al., 2009; Satapanajaru et al., 2011).

### Exoelectrogenic Behaviors of Strain KVM11

#### Current Generation in Microbial Electrochemical Cells Fed With Acetate and Citrate

**FIGURE 5:**
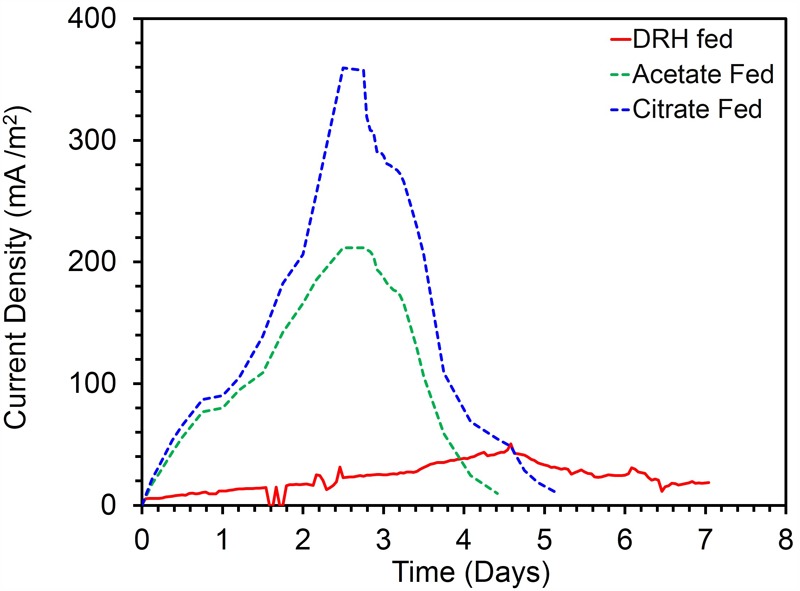
Current production by *Citrobacter* sp. KVM11 in single chamber MFCs containing brush anodes. Green dotted line shows a representative cycle of current production in acetate (1g/L) fed MFCs; Blue dotted line shows a representative cycle of current production in citrate (1g/L) fed MFCs; Red dotted line shows a represntative cycle of current production in diesel range hydrocarbons (DRH) (800mg/L).

**FIGURE 6:**
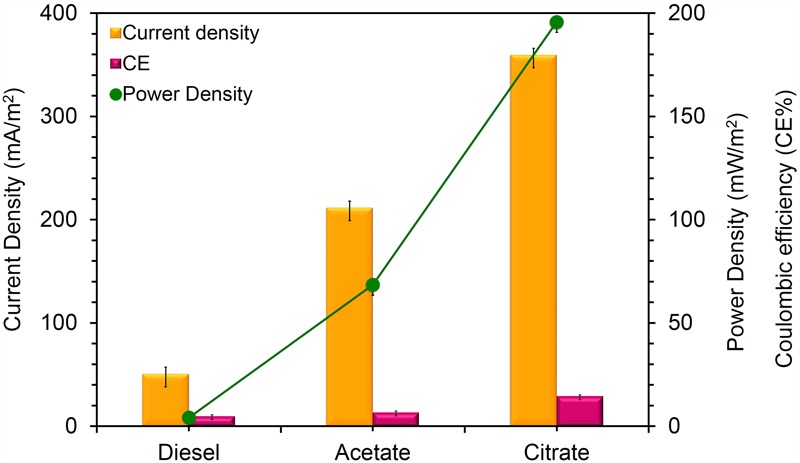
Current density, Power density and Coulombic efficiency relationship for MFCs fed with different substrates. Yellow bars represents the current density produced from the oxidation of acetate, citrate and DRH; Pink bars represent the coulombic efficiency of each MFC system fed with acetate, citrate and DRH; Green line represents power density generation from the oxidation of acetate, citrate, and DRH.

**FIGURE 7:**
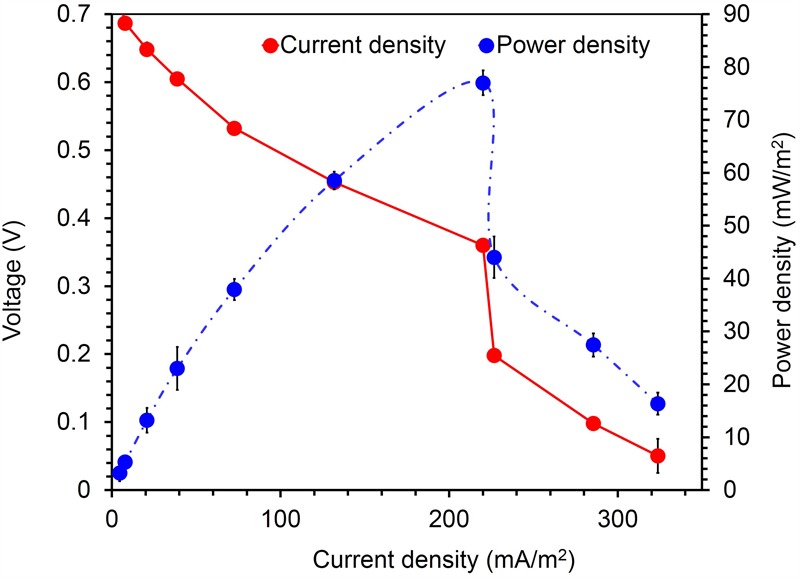
Current- voltage and current-power relationship for MFCs fed with acetate (1g/L) *Citrobacter* sp. KVM11. Red circles represent the current density produced from the oxidation of acetate in MFCs; Blue circles represent the power density generated from the oxidation of acetate in MFCS.

**FIGURE 8:**
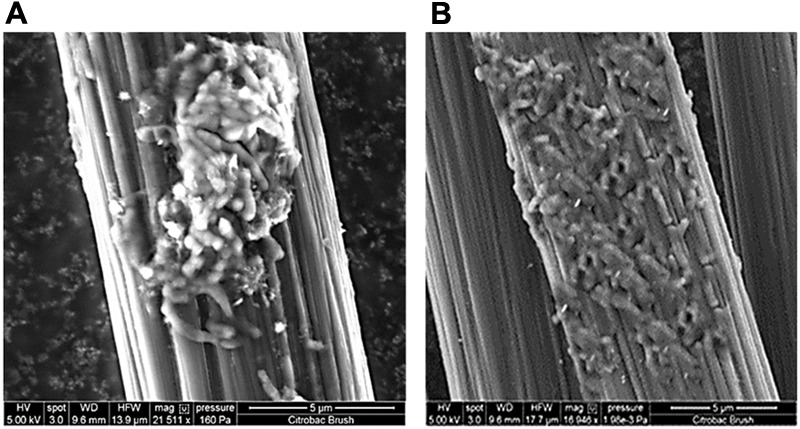
Electrode micrographs: **(A)** New, uninoculated control anode **(B)** SEM micrograph of an electrode surface following growth of *Citrobacter* sp. KVM11 biofilm with acetate (1g/L) as an electron donor under MFC conditions.

To confirm the extracellular access to the insoluble electron acceptors, the exoelectrogenic properties of the strain KVM11 were investigated in three different conditions (i) acetotrophic (ii) dye decolorization, and (iii) hydrocarbonoclastic, using microbial electrochemical systems. To initiate bacterial growth on a brush electrode, cells of exponential phase cultures grown in Fe(III) citrate (10 mM) were inoculated in an anodic chamber of a single chamber MFC. Upon inoculation with strain KVM11, the anodic current was generated within a few hours using sodium acetate (20 mM) as a sole carbon source. During the first cycle, the voltage was steadily increased, and a maximum open circuit voltage between the electrodes was 0.720 ± 25 mV. Once the voltage stabilized, the electrodes were connected through a fixed resistance (1000 ω). The present study exhibited a maximum power density of 212 mW/m^2^ (**Figure 5**) with a recovery of 13.3% (**Figure 6**) as an electrical current using the strain KVM11 in acetotrophic conditions. Such findings suggest that the strain KVM11 is also capable of utilizing insoluble electron acceptors with the additional distinctive features making this strain unique from its counterparts (Xu and Liu, 2011; Huang et al., 2015; Liu et al., 2016), which include, (i) ability to degrade PH, (ii) dye detoxification in MERS, and (iii) anoxic Fe(III) reduction. The typical polarization curve of *Citrobacter* strain KVM11 (**Figure 7**) in an acetate-fed MFC indicated a large potential drop due to activational losses as shown in other exoelectrogenic strains (Park et al., 2001; Pham et al., 2003; Venkidusamy and Megharaj, 2016a,b). Scanning electron micrographs (SEM) from the final cycle electrode revealed its colonization by bacteria, forming multilayers of a thick biofilm around the electrode surface (**Figure 8**). Current generation by strain KVM11 also examined using sodium citrate as their sole carbon source at the same external resistance. When the repeatable and stable performance of current output was achieved, citrate fed MFC generated the maximum power density of 195.82 mW/m^2^ at a current density of 359.64 mA/m^2^. The overall calculated CE was 28.9% (**Figure 6**). Regardless of the types of substrates tested, *Citrobacter* sp. KVM11 produced anodic currents (acetate 212 ± 3 mA/m^2^; citrate 359 ± 4 mA/m^2^) in MFCs. These experimental results supported the fact that Fe(III) reducing *Citrobacter* sp. strain KVM11 can be used as an exoelectrogen in microbial electrochemical systems as previously described in *Citrobacter* sp. LAR-1 (Liu et al., 2016).

#### Electron Transfer Mechanism of Strain KVM11

**FIGURE 9:**
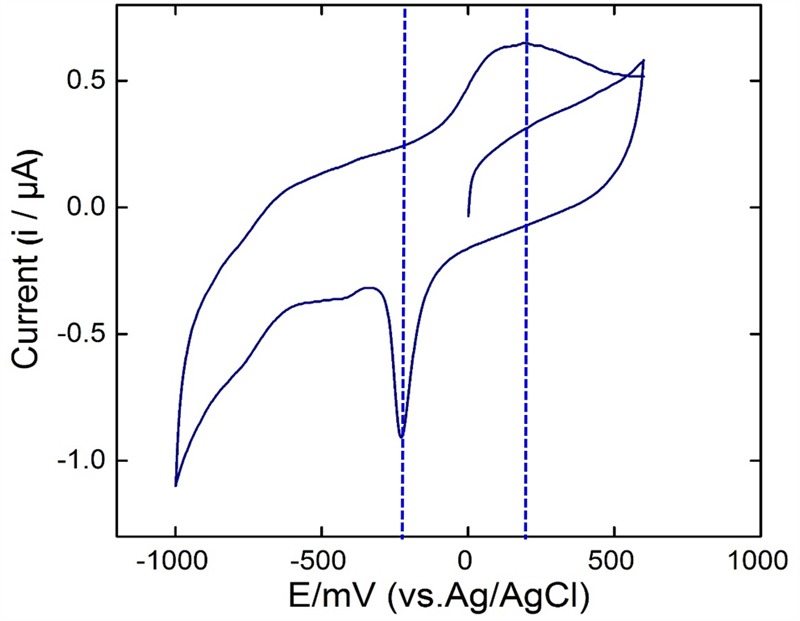
Cyclic voltammetry studies. Voltammograms of the bacterial suspension prepared from anaerobically grown cells of *Citrobacter* sp. KVM11. The scan rate was 5 mV s^-1^ with a potential range from -800 to 800 mV.

To further investigate the exoelectrogenic nature of the strain KVM11, the interaction between electrodes and cell suspensions on glassy carbon electrodes was observed. Cell suspensions of the strain were prepared from Fe(III) (10 mM) supplemented cultures and their electrochemical activities examined in respective aerobic and anaerobic conditions as stated earlier (Park et al., 2001; Pham et al., 2003). According to the voltammograms, the anaerobically grown cells of strain KVM11 showed electrochemical activity through the presence of a reduction peak ranging from -100 to -310 mV and oxidation peaks at the range of +100 mV to +300 mV observed against the Ag/AgCl reference electrode (**Figure 9**). The asymmetry of CV peaks at different potentials (-800 to 800 mV) indicates that the reaction is a quasi-reversible reaction. One redox couple was observed from the CV peak, and a number of electrons (*e* = 1) transferred was calculated from the Nernst equation (Logan, 2008). The mid-potential of the CV peaks showed – 205 mV, which is characteristic of the c-type cytochromes as reported earlier (Myers and Myers, 1992; Kim et al., 1999). For example, the midpoint potentials of OmcA (c-type cytochromes) reported in *Shewanella* MR-1 biofilm were -201 and -208 mV (Kim et al., 1999). No obvious redox pair peak was observed from the suspension of aerobically grown cells or autoclaved controls. The results also perhaps indicated that oxygenated liquid cultures prevent the synthesis of the outer membrane cytochromes which plays a major role in electron transfer mechanisms (Kim et al., 2002).

### Electrobioremediation Potential of Strain KVM11

#### Energy Generation in Hydrocarbon Fed MERS

**FIGURE 10:**
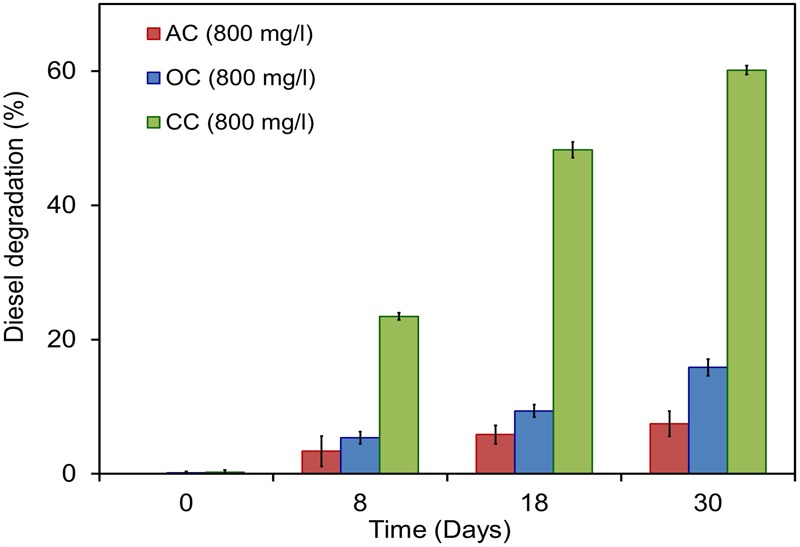
Electrobioremediation of DRH by *Citrobacter* sp. KVM11. Pink bar represents the percent of hydrocarbon removal in abiotic control samples containing 800 mg/l of DRH; Blue bar represents the percent of hydrocarbons removal in open circuit reactors containing 800 mg/l of DRH; Green bar represents the percent of hydrocarbons removal in closed circuit reactors containing 800 mg/l of DRH.

*Citrobacter* sp. have been widely examined for its biormediation potential because of its wide spectrum use of various xenobiotic pollutants (Macaskie et al., 1995; Narde et al., 2004; Qiu et al., 2009, 2017; Wang et al., 2017). Members of this genus have been found along with other predominant genera of hydrocarbon degraders including *Acinetobacter, Pseudomonas, Alcaligenes*, and *Sphingomonas* in oil-contaminated environments as stated earlier (Chikere et al., 2012). Recent studies on the electrochemically mediated degradation of hydrocarbons demonstrated the presence of *Citrobacter* sp. in the anodic microbial communities (Morris et al.,2009; Venkidusamy et al., 2015). The capability of hydrocarbon mineralization by *Citrobacter* sp. has been demonstrated earlier only under aerobic conditions (Singh and Lin, 2008; AlDisi et al., 2016). However, the bioremediation of these compounds in anaerobic environments or MERS by the genus *Citrobacter* is previously unknown. In the present study, experiments were conducted to examine the exoelectrogenic property of the strain KVM11 using DRH (concentration of 800 mg l^-1^) as a sole carbon source in hydrocarbon fed MERS systems for five consecutive runs. A maximum current and power density obtained at this concentration were 50.64 ± 7 mA/m^2^, 4.08 ± 2 mW/m^2^ (**Figure 6**). An average of 60.14 ± 0.67% DRH removal with the simultaneous electrical current recovery of 9.6% (**Figure 6**) was observed in closed circuit MERS (**Figure 10**) at the end of the batch studies (30 days). In the case of the abiotic (AC) and open circuit (OC) controls, DRH removal rates were low (7.45 ± 1.99%, 15.84 ± 1.23%, respectively) by the end of the batch study. An increase in current (70.57 mA/m^2^) and power densities (15.04 mW/m^2^) were observed in a similar device through earlier investigations (Venkidusamy et al., 2015) on mixed culture hydrocarbonoclastic enrichments suggesting that the higher rate of substrate assimilation (83 ± 2.6%) in hydrocarbons fed MERS. Similar effects have been shown by other authors using different substrates including recalcitrant contaminants (Luo et al., 2009; Shen et al., 2012; Hassan et al., 2016). This perhaps indicates the presence of a complex ecology in the mixed culture hydrocarbonoclastic enrichments and its synergistic effects between hydrocarbonoclastic and exoelectrogenic bacterial groups in hydrocarbon fed MERS systems. Therefore, enrichment of selective bacterial population is needed during the bioaugmentation process of MERS systems to enhance the contaminant degradation with simultaneous energy gain.

#### Energy Generation in Dye Fed MERS

**FIGURE 11:**
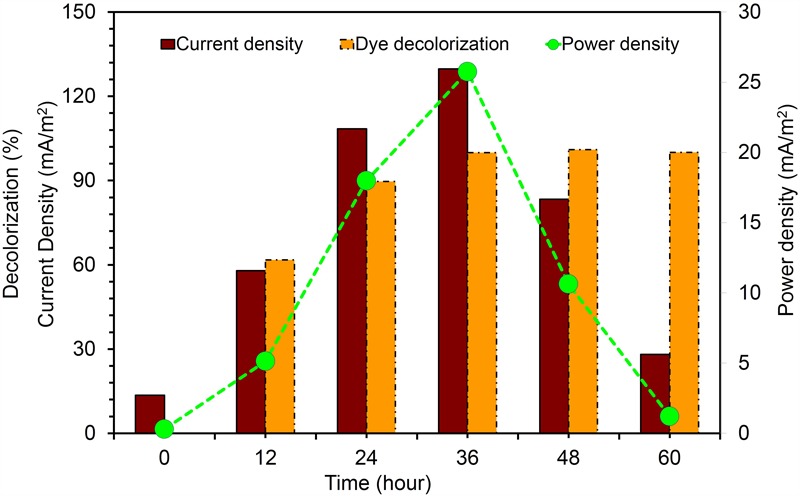
Current generation and dye detoxification by *Citrobacter* sp. KVM11 in azo dye fed MERS Brown bar represents the current density produced from RB5 (400 mg/l) decolorization. Yellow bar represents the percent of RB5 removal in dye fed (400 mg/l) MFCs. Green closed circles represent the power density generation fron RB5 (400 mg/l) fed MFCs.

The current was rapidly generated in all dye fed MFCs inoculated with KVM11 cells within few hours of using azoic dye as an energy source at a fixed resistance of 1000 ω. The maximum output range of voltage, current density, and power density were 200 ± 2 mV, 125.9 ± 4 mA/m^2^, and 25 ± 0.75 mW/m^2^, respectively. Constant and repeatable power cycles were obtained during four changes of the anolytes of the anode chamber. Using RB5 concentration of 400 mg l^-1^ in MFC, 61.74% was removed during the first 12 h of operation. After 24 h, almost 89.60% of RB5 was decolorized, and it was below detection limits at the end of the batch operation (60 h) as shown in **Figure 11**. Recent investigations have revealed the potential of using such pure cultures of heterotrophic biofilms in MERS for dye detoxification (Chen et al., 2010a,b). For example, Chen et al. (2010a), reported the possibility of using pure cultures of *Proteus hauseri* in MFC, however, decolorization efficiency and power densities generated were much lower. The performance of these microbial electrochemical cells using pure cultures of exoelectrogens for dye detoxification were considerably affected by a number of reactor parameters, operating conditions and efficacy of bacterial strains used as reported earlier (Min et al., 2005; Logan, 2008).

In summary, the findings presented in our study suggest that the strain KVM11 is capable of utilizing solid electron acceptors through extracellular transfer mechanisms without the addition of external mediators. The biodegradation experiments showed the evidence for the existence of electrochemical mediated degradation capability in *Citrobacter* sp. KVM11 in MERS conditions. An increasing number of studies demonstrate the potential use of facultative anaerobes in MERS for enhanced degradation of recalcitrant contaminants (Morris et al., 2009; Huang et al., 2011; Yan et al., 2012; Venkidusamy et al., 2015; Venkidusamy and Megharaj, 2016a,b). However, the optimization of such contaminant degrading mechanism will require a deeper understanding at molecular level through proteomics or transcriptomics of those microbial candidates.

## Conclusion

Based on molecular and metabolic characterization, we identified that the strain KVM11 obtained from MERS represents a novel strain that is phylogenetically related to *Citrobacter* sp. A dissimilatory reduction of Fe(III) by strain KVM 11 confirmed the possibilities of using iron reducers in microbial electrochemical systems as previously described (Liu et al., 2016). While the exoelectrogenic metabolism of this species is previously known (Xu and Liu, 2011; Liu et al., 2016), our present findings demonstrated for the first time the bioelectrochemical degradation of hydrocarbons and its associated electrochemical properties. Identification of such organisms from MERS expands the known diversity of exoelectrogens and provides the novel strain to explore the three-way interaction between microbe-electrode-contaminant through EET mechanism. Also, bioelectrochemically mediated detoxification of diaozic dye by strain KVM11 reveals its potential for application in the treatment of waste from textile industries using MERS. Altogether, our findings reflect the metabolic versatility of *Citrobacter* sp. KVM11 which holds promise in the bioelectrochemical remediation of recalcitrant xenobiotics with simultaneous energy generation, in the form of electricity. Further, electromicrobiological studies show the potential of unfolding molecular mechanism of complex interactions of *Citrobacter* sp. KVM11 between solid phase electron acceptors and contaminants. The availability of complete genome and its analysis will provide more details about the functions of this bacterium.

## Author Contributions

KV and MM proposed the study. KV conducted the experiments under the supervision of MM. KV and ARH analyzed the data and prepared the draft with contributions from MM.

## Conflict of Interest Statement

The authors declare that the research was conducted in the absence of any commercial or financial relationships that could be construed as a potential conflict of interest. The reviewer GZ and handling Editor declared their shared affiliation.

## Footnotes

1 http://www.ncbi.nlm.nih.gov
