# Supplementary material for: Petrophilic, Fe(III) Reducing Exoelectrogen Citrobacter sp. KVM11, Isolated From Hydrocarbon Fed Microbial Electrochemical Remediation Systems
Source: Front Microbiol. 2018 Mar 12;9:349. doi: 10.3389/fmicb.2018.00349 (PMC5858583; doi:10.3389/fmicb.2018.00349)

Supplementary Material

Petrophilic, Fe(III) reducing exoelectrogen *Citrobacter sp.* KVM11, isolated from hydrocarbon fed microbial electrochemical remediation systems

Krishnaveni Venkidusamy[a,b,*], Ananda Rao Hari [d] , Mallavarapu Megharaj[a,b,c]

Correspondence: Dr. Krishnaveni Venkidusamy, krishnaveni.venkidusamy@mymail.unisa.edu.au

Supplementary Figures

Figure S1: Single-chamber microbial fuel cell configuration. WE-working electrode (graphite brush); RE- reference electrode R-resistor; V-voltage

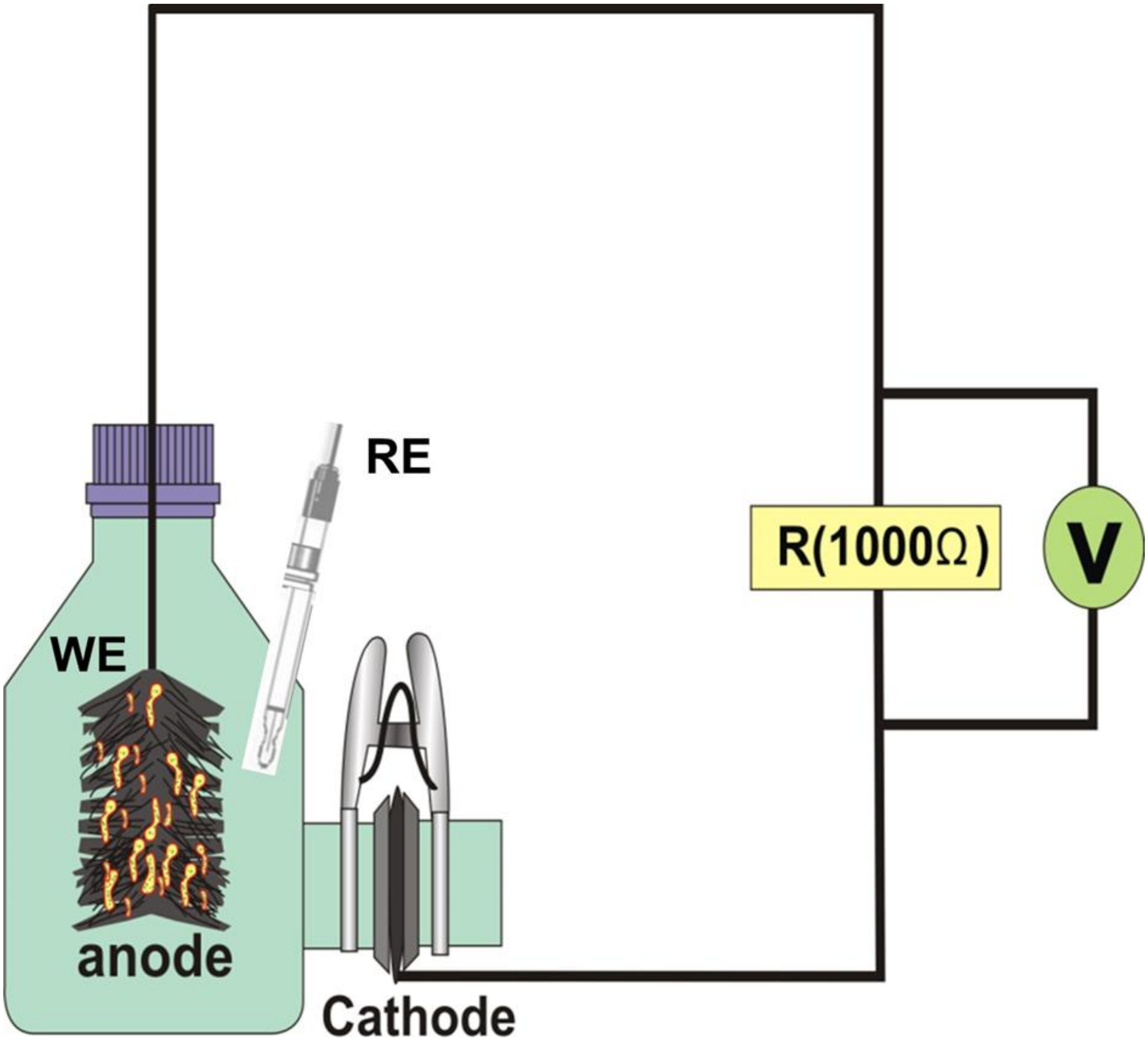

Supplement: Supplementary file 1 [file Image_1.PDF]
